# Brain Plasticity in Blind Subjects Centralizes Beyond the Modal Cortices

**DOI:** 10.3389/fnsys.2016.00061

**Published:** 2016-07-08

**Authors:** Laura Ortiz-Terán, Tomás Ortiz, David L. Perez, Jose Ignacio Aragón, Ibai Diez, Alvaro Pascual-Leone, Jorge Sepulcre

**Affiliations:** ^1^Department of Neurology, Division of Cognitive Neurology, Berenson-Allen Center for Noninvasive Brain Stimulation, Beth Israel Deaconess Medical Center, Harvard Medical SchoolBoston, MA, USA; ^2^Department of Radiology, Division of Nuclear Medicine and Molecular Imaging, Massachusetts General Hospital, Harvard Medical SchoolBoston, MA, USA; ^3^Gordon Center for Medical Imaging, Massachusetts General HospitalBoston, MA, USA; ^4^Departamento de Psiquiatría, Facultad de Medicina, Universidad Complutense de MadridMadrid, Spain; ^5^Department of Neurology, Massachusetts General Hospital, Harvard Medical SchoolBoston, MA, USA; ^6^Department of Psychiatry, Massachusetts General Hospital, Harvard Medical SchoolBoston, MA, USA; ^7^Athinoula A. Martinos Center for Biomedical Imaging, Massachusetts General Hospital, Harvard Medical SchoolCharlestown, MA, USA; ^8^Departamento de Radiodiagnóstico, Hospital Universitario Puerta de Hierro de MajadahondaMadrid, Spain; ^9^BioCruces Health Research Institute, Cruces University HospitalBarakaldo, Spain

**Keywords:** congenital blind, fMRI, functional connectivity, late-onset blind, multimodal integration network

## Abstract

It is well established that the human brain reorganizes following sensory deprivations. In blind individuals, visual processing regions including the lateral occipital cortex (LOC) are activated by auditory and tactile stimuli as demonstrated by neurophysiological and neuroimaging investigations. The mechanisms for such plasticity remain unclear, but shifts in connectivity across existing neural networks appear to play a critical role. The majority of research efforts to date have focused on neuroplastic changes within visual unimodal regions, however we hypothesized that neuroplastic alterations may also occur in brain networks beyond the visual cortices including involvement of multimodal integration regions and heteromodal cortices. In this study, two recently developed graph-theory based functional connectivity analyses, interconnector analyses and local and distant connectivity, were applied to investigate functional reorganization in regional and distributed neural-systems in late-onset blind (LB) and congenitally blind (CB) cohorts each compared to their own group of sighted controls. While functional network alterations as measured by the degree of differential links (DDL) occurred in sensory cortices, neuroplastic changes were most prominent within multimodal and association cortices. Subjects with LB showed enhanced multimodal integration connections in the parieto-opercular, temporoparietal junction (TPJ) and ventral premotor (vPM) regions, while CB individuals exhibited increased superior parietal cortex (SPC) connections. This study reveals the critical role of recipient multi-sensory integration areas in network reorganization and cross-modal plasticity in blind individuals. These findings suggest that aspects of cross-modal neuroplasticity and adaptive sensory-motor and auditory functions may potentially occur through reorganization in multimodal integration regions.

## Introduction

Cross-modal neuroplasticity has been proposed as a fundamental mechanism by which individuals without sight recruit visual-related cortices to process sensory information from other perceptual sensory modalities (Amedi et al., 2003; Merabet et al., 2004; Bedny et al., 2011; Ortiz et al., 2011; Watkins et al., 2013). Functional imaging studies in late-onset blind (LB) and congenitally blind (CB) subjects have shown that, regardless of task and age of onset, there is consistent activation of brain areas such as the lateral surface of the occipital cortex (LOC) during auditory and/or tactile sensory tasks. These findings suggest cross-modal plasticity in visual-related cortices (Amedi et al., 2003; Bedny et al., 2011) in blind subjects, as many studies have shown using whole brain task analyses with different functional neuroimaging techniques (Supplementary Figure S1 shows the main activation foci of previous cross-modal, task-based studies that used a whole-brain analytical approach). In this context, cross-modal visual cortex activations have often been interpreted as a cortical mechanism to substitute de-afferented functions or to facilitate higher-order cortical integration with other sensory modalities (Amedi et al., 2003; Merabet et al., 2004; Bedny et al., 2011; Ortiz et al., 2011; Collignon et al., 2013; Watkins et al., 2013). Indeed, in CB individuals, increasing occipital cross-modal activation is associated with superior tactile discrimination abilities, better sound localization capacities, and even greater verbal memory (Amedi et al., 2003). It remains unclear, however, if these areas act in isolation or as part of more broadly distributed neuroplastic alterations occurring across brain networks. Notably, although many interconnectivity changes have been observed in the LOC, cortical activations in other regions have also been described. For example, reorganization of functional connections between the perceptual cortex and heteromodal areas have been recently reported (Bedny et al., 2011; Burton et al., 2014).

Mesulam (1998) postulated that sensory information undergoes extensive associative elaboration and attentional modulation as it becomes incorporated with other aspects of cognition. It has been previously suggested that this process occurs along a core synaptic hierarchy, with reciprocal connections from one zone to another and with higher synaptic levels exerting a top-down influence upon earlier processing levels. More recently, graph theory based analyses applied to blood-oxygen-level-dependent (BOLD) signal fMRI data have allowed for the *in vivo* identification of relay stations that bridge unimodal systems to networks engaged in higher-order cognitive functions (Sepulcre et al., 2012). Thus far, very few studies in the field of blindness have used whole brain functional connectivity analyses, and the ones that have done so have not used graph theory approaches (Liu et al., 2007). For example, independent component analyses in resting state fMRI have been applied to CB subjects compared to controls demonstrating among other findings enhanced connectivity between the salience and frontoparietal control networks (Wang et al., 2014). Task-based fMRI has been previously well implemented to study cross-modal plasticity (Pascual-Leone et al., 2005; Merabet and Pascual-Leone, 2010). For these reasons, we applied graph theory methods to detail network reorganization and characterize the functional connectivity relationships between unimodal regions, multimodal integration areas and association cortices in LB and CB individuals compared to sighted controls.

While there are several models conceptualizing the hierarchical cortical structure of the brain, one model suggests that the human brain network architecture goes from primary unimodal cortex to multimodal regions, and ultimately to associative areas that support high-order cognitive functions (Sepulcre et al., 2012). Multimodal areas are particularly relevant to the understanding of higher-order cognitive functions such as the construction of an integrated, multisensory perceptual experience (Sepulcre et al., 2010). Cortical hubs are a distinct set of regions that merge the highest number of functional distributed connections in the human brain, leading to the interpretation of these regions as the top hierarchical areas of brain integration.

In this study, we specifically aimed to broaden the identification of neuroplastic network reorganization, focusing on the relationship between multimodal and primary cortical regions, across both visual and non-visual systems in CB and LB subjects. For these purposes, we used functional connectivity magnetic resonance imaging (fcMRI) and graph theory to assess differences in regional and large-scale distributed functional networks in CB and LB subjects compared to sighted individuals. fcMRI is a neuroimaging technique that uses spontaneous fluctuations in low-frequency BOLD signal to investigate functional (temporal) coupling between brain regions (Biswal et al., 1995; Greicius et al., 2003). Functional connectivity patterns have been shown to be complex and region-specific, with for instance high local modularity in primary sensory cortices and widespread distant connections in high-order processing areas such as multimodal and association cortices (Jones and Powell, 1970; Mesulam, 1990, 2008; Felleman and Van Essen, 1991; Ungerleider and Haxby, 1994; Salvador et al., 2005; Buckner et al., 2009; Sepulcre et al., 2010). Graph theory disentangles these network patterns by providing an analytical framework delineating critical nodes and links within and across distributed networks (Sporns, 2013). The main methods used in this study, *Interconnector Analysis* (Sepulcre et al., 2012) and *Local and Distant Connectivity Analysis* (Sepulcre et al., 2010), are recently developed techniques that characterize the convergence and interactions of brain systems at the connectivity level. *Interconnector Analysis* enables the interrogation of functional communications between perceptual systems and higher-order brain systems in blind subjects. Functional connectivity profiles between unimodal sensory cortices (visual, auditory and somato-motor), multimodal integration areas (i.e., lateral occipitotemporal, dorsal anterior cingulate and superior parietal cortices (dACCs and SPCs); Sepulcre et al., 2012) and regions of higher-order, distributed cognitive neural-systems known as cortical hubs (i.e., precuneus, middle and inferior temporal gyri; Buckner et al., 2009; Sepulcre et al., 2010) were investigated. The *Local and Distant Connectivity* data-driven methods were also used to quantify prominent centers of connectivity across the whole brain.

We hypothesized that cortical functional connectivity in blind subjects compared to sighted-individuals would reflect neuroplastic changes not only in unimodal sensory cortices, but also in distributed multimodal and association brain areas which are known to function as a bridge allowing for sensory integration across modalities. Our hypotheses leverage the ability to examine broadly distributed network relationships, that can be particularly disentangled using graph-theory based multivariate network analyses which have been minimally applied to the study of neuroplastic changes in blind subjects to date. We also hypothesized that LB subjects in relation to CB subjects each compared to their own sighted controls would show distinct patterns of network reorganization given that LB subjects had prior visual perceptual abilities. Specifically, we hypothesized that CB subjects would demonstrate alterations in earlier connectional axes of the visual and multimodal integration streams, while individuals with later onset blindness would show more upstream alterations in multimodal processing areas (Sepulcre et al., 2012). Our findings support this hypothesis, and advance our understanding of cortical centers of functional connectivity and network reorganization in LB and CB individuals.

## Materials and Methods

### Study Participants

Seven LB subjects (mean age: 45.1 ± 10.1; M/F: 5/2; mean head motion: 0.06 mm) and 11 CB subjects (mean age: 44.2 ± 15.3; M/F: 6/5; mean head motion: 0.27 mm) were included in the study analyses (Table 1). LB subjects were recruited from the Spanish National Organization of the Blind and all had lost their vision after 14 years of age (Cohen et al., 1999; Sadato et al., 2002). Causes of blindness included: glaucoma, retinal necrosis, retinal detachment, and retinitis pigmentosa amongst other etiologies. None of the LB subjects had any remaining visual light perception; three of the CB subjects had minimal light perception. The CB subjects were recruited through the Brain and Cognitive Sciences Department at the Massachusetts Institute of Technology (Deen et al., 2015). The cause of their blindness was mainly retinopathy of prematurity and different pathologic conditions of the optic nerve. None of the participants had any additional neurological or psychiatric history.

**Table 1:** **Demographic and clinical information for congenital blind (CB) and late-onset blind (LB) subjects**.

Subject	Age	Gender	Cause of blindness	Onset of vision loss	Handedness	Residual light perception
**CB1**	58	M	Retinopathy of Prematurity	Birth	R	No
**CB2**	41	M	Unknown nerve disconnection between eyes and brain	Birth	L	No
**CB3**	41	M	Retinoblastoma	Birth	R	No
**CB5**	64	F	Optic nerve malformation	Birth	R	Minimal
**CB6**	30	M	Optic nerve hypoplasia	Birth	L	Minimal
**CB7**	32	M	Retinopathy of Prematurity	Birth	L	No
**CB8**	62	F	Retinopathy of Prematurity	Birth	R	Minimal
**CB9**	57	F	Retinopathy of Prematurity	Birth	R	No
**CB10**	62	F	Retinopathy of Prematurity	Birth	R	No
**CB11**	25	F	Retinopathy of Prematurity	Birth	R	No
**CB12**	24	M	Anophthalmia	Birth	R	No
**LB1**	55	F	Glaucoma	48	R	No
**LB2**	31	M	Retinitis Pigmentosa	16	R	No
**LB3**	47	M	Iatrogenic	30	R	No
**LB4**	59	F	Retinitis Pigmentosa	45	R	No
**LB5**	44	M	Retinal Necrosis	22	R	No
**LB6**	46	M	Glaucoma	20	R	No
**LB7**	34	M	Retinal Detachment	14	R	No

For comparison, we studied two separate healthy control groups of sighted participants matched to the blind subjects in age, gender and mean MRI head movement (7 LB control subjects: mean age 45.2 ± 9.9; M/F: 5/2; mean motion: 0.08 mm (*p* = 0.26); 12 CB control subjects: 42.8 ± 13.6; M/F: 6/6; mean motion: 0.25 mm (*p* = 0.25)). Of note, LB and CB subjects were not directly compared due to their distinct imaging acquisition protocols and, more importantly, because head movement was significantly different across LB and CB groups (*p* < 0.001). Differences in head motion between study groups can introduce aberrant functional connectivity results that impact the validity and interpretation of the findings (Van Dijk et al., 2010; Power et al., 2014).

All participants were informed of the nature of the experiment and provided written informed consent prior to participation in accord with the Declaration of Helsinki. Institutional review board approval was obtained from the Massachusetts Institute of Technology and the Hospital Universitario Clínico San Carlos of the Universidad Complutense de Madrid.

### Data Acquisition

LB subjects and their matched controls (MCs) were scanned on a 3 Tesla (ACHIEVA 3.0T TX, Philips) MRI scanner using an 8-channel phased-array head coil. High-resolution 3D T1-weighted magnetization prepared rapid acquisition gradient echo (T1W 3D TFE SENSE) images were acquired for anatomic reference (TR = 7.6 ms, TE = 3.5 ms, FA = 7°, 1.0 mm isotropic voxels). Functional data was acquired using an echo planar imaging (EPI) pulse sequence sensitive to BOLD contrast (TR = 3000 ms, TE = 30 ms, FA = 90°, 3.0 mm isotropic voxels), each run lasted 6 min 15 s. CB subjects and their MCs were scanned on a 3 Tesla (Siemens 3.0T Magnetom Trio) MRI scanner using an 8-channel phased-array head coil. Anatomical data were collected using a MPRAGE pulse sequence (TR = 2 ms, TE = 3.39 ms, FA = 9°, 1.33 mm isotropic voxels). Functional data were acquired using an EPI pulse sequence sensitive to BOLD contrast (TR = 6000 ms, TE = 30 ms, FA = 90°, 2 × 2 × 2 mm voxels), each run lasted 6 min 24 s. In all cases, head motion was restricted using a pillow and several foams, and earplugs were used to attenuate scanner noise. During the functional runs, participants were asked to stay awake with their eyes open for each of the BOLD runs and remain as still as possible.

### Functional Connectivity Preprocessing Analysis

Resting-state MRI analysis procedures were optimized for fcMRI analyses (Biswal et al., 1995). First, images were reoriented and the first four volumes were removed to allow T1-equilibration. Slice-acquisition-dependent time shifts were corrected per volume, followed by head motion correction and normalization to the Montreal Neurological Institute (MNI) atlas space (Statistical Parametric Mapping (SPM2), Wellcome Department of Cognitive Neurology, London, UK) to yield a volumetric time series resampled at 2 mm cubic voxels. Data were spatially smoothed using a 4 mm FWHM Gaussian blur to increase the signal to noise ratio (SNR) and a bandpass filter was introduced to retain frequencies below 0.08 Hz. Several sources of nonspecific variance were removed by regression of nuisance variables including head motion and the signals averaged over the whole-brain, the lateral ventricles and a region centered in the deep cerebral white matter. By removing global signal, variance contributed by physiological artifacts was minimized. Removal of signals correlated with ventricles and white matter further reduced non-neuronal contributions to BOLD correlations (Van Dijk et al., 2010). Finally, for computational efficiency, down-sampling the data to 8 mm isotropic voxels was performed (Sepulcre et al., 2010).

### Statistical Preprocessing Analysis

Prior to graph theory analyses, the following steps were performed at the individual-subject level on the fcMRI data: (I) the Pearson R correlation coefficient, or product-moment correlation coefficient, was computed between pairs of voxels across the whole brain using the time course low-frequency BOLD fluctuations and a brain mask of 5138 voxels (*n*) in order to obtain a final *n × n* association matrix for each individual. (II) Only positive correlations were included in all analyses due to the ambiguous origin and interpretation of negative correlations (Van Dijk et al., 2010), for this reason all numbers in the matrix below 0 were taken as not-a-number for computation purposes. (III) Lastly, a second level statistical parametric analysis (two sample *t*-test, Matlab v8.0, The Mathworks, Inc., Natick, MA, USA) was used to obtain the functional differential links between blind and matched control groups (blind > sighted and sighted > blind). As a final step before the graph theory analyses, *t*-test matrices were corrected for multiple comparisons using their associated *p*-values and a False Discovery Rate (FDR) correction (Benjamini and Hochberg, 1995) at *q*-level of 0.05 to control for the rate of false positives. The resulting corrected *t*-test matrices were then binarized and served as graph input for all graph theory analyses of the study (Supplementary Figures S2, 3).

#### Functional Connectivity Postprocessing Analysis

##### Connectivity Within and Between Cortical Systems

In order to describe both the enhanced and the disrupted connectivity in blind individuals, the differential functional connections between blind subjects and controls were specifically characterized using a graph theory method described previously (Sepulcre et al., 2012; Sepulcre, 2015), called *Interconnector Analysis*. This method uses brain graphs to compute the number of connections that a given set of nodes (defined by mask *a*) has to another set of nodes (defined by mask *b*). The computation was done at the node level, such that for each node of mask *a* all of its links, or degree connectivity, to nodes in mask *b* were calculated. The *Interconnector Analysis* also provides the reverse computation, that is, the degree of connectivity of nodes in mask *b* to mask *a*, obtaining “between” systems connectivity profiles. Moreover, if mask *a* and *b* are the same mask, then it provides the “within” system connections. The *Interconnector Analysis* is formally described as follows:

(1) $$DDL(a,b)=\sum_{i=1}^{m}\sum_{j=1}^{n}d(i,j)$$

where *i* represents the voxels in mask *a*, *j* the voxels in mask *b* and *m* and *n* the number of voxels in mask *a* and *b* respectively. Finally *d*(*i, j*) is equal to 1 if the blind subjects have higher number of significant links compared to controls (or the reverse statistical contrast), based on a *t*-test analysis with FDR correction and 0 otherwise.

In summary, we used the number of differential functional connections between blind and sighted groups, also referred to as degree of differential links (DDL), as inputs for the *Interconnector Analysis*. Therefore, all computations were calculated by counting in the FDR-corrected *t*-test matrices the differential links occurring between two selected masks of interest. The sensory related masks were chosen from the 17 brain parcellations performed by Yeo et al. (2011) based on the intrinsic functional connectivity of the human cerebral cortex from 1000 subjects and adapted for these analyses.

**Figure 1:**
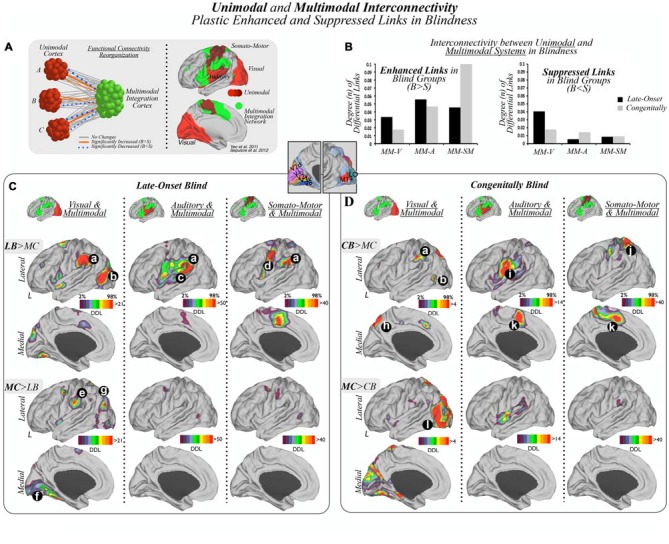
**Unimodal and multimodal interconnectivity plastic enhanced and suppressed links in blindness. (A)** Unimodal cortices (visual, auditory and somato-motor), represented as different shades of red, as well as the multimodal integration network, displayed in green, were used as *a priori* regions of interest, in both late-onset blind (LB) and congenitally blind (CB) subjects compared to their matched sighted controls (MC), to characterize enhanced or suppressed interconnectivity between them. B = Blind, S = Sighted. **(B)** Bar graphs show statistically significant increased or enhanced (left) and decreased or suppressed (right) interconnectivity between multimodal integration regions and unimodal cortices in blind subjects compared to matched controls (MCs). Results in bar graphs show the total amount of false discovery rate (FDR)-corrected functional connections that differ between studied groups, and, thus, standard deviation or error bars do not apply here. V = visual, A = auditory, SM = somato-motor, MM = multimodal. Panels **(C,D)** present interconnectivity patterns in LB and CB subjects compared to their MCs respectively (visual and multimodal, auditory and multimodal and somato-motor and multimodal). Panels **(C,D)** (a) temporo-parietal junction (TPJ)/supramarginal gyrus (SMG); (b) lateral occipital cortex (LOC); (c) posterior aspect of the superior temporal gyrus; (d) motor cortex; (e) operculum parietale (OP); (f) medial aspects of the lingual and fusiform gyrus; parieto-occipital junction (g); (h) precuneus; (i) ventral areas of the somato-motor cortex; (j) superior parietal cortex (SPC); (k) medial supplemental motor area (SMA); and (l) occipital lobe. Note, in Panels **(C,D)** relative color scales were used to aid visualization, however, visual-multimodal region analyses had notably lower degree of connectivity changes compared to auditory or somato-motor analyses.

**Figure 2:**
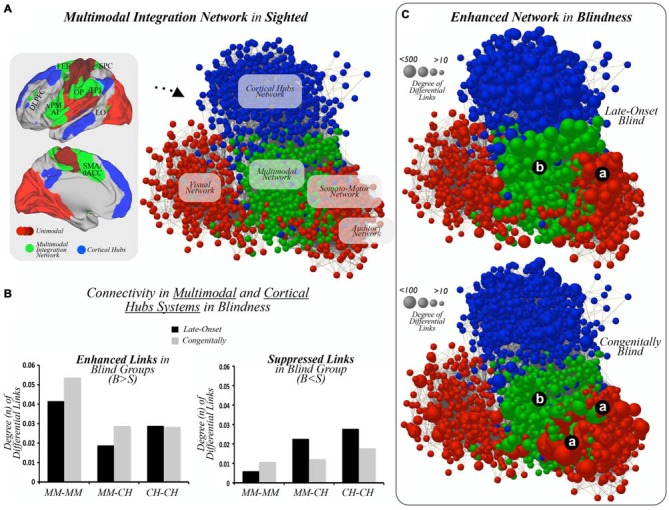
**Multimodal integration networks. (A)** Unimodal sensory cortices (different shades of red), multimodal integration cortices (green), and cortical hubs (blue) are displayed on template brains, as well as on a graph layout illustrating connectivity patterns across systems in the sighted controls. DLPFC = dorsolateral prefrontal cortex, FEF = frontal eye field, SPC = superior parietal cortex, vPM = ventral premotor, AI = anterior insula, OP = operculum parietale, TPJ = temporo-parietal junction, LO = lateral occipital, dACC = dorsal anterior cingulate cortex. **(B)** Bar graphs show statistically significant increased or enhanced (left) and decreased or suppressed (right) interconnectivity between multimodal regions and cortical hubs in blind subjects compared to MCs. Results in bar graphs show the total amount of FDR-corrected functional connections that differ between studied groups, and, thus, standard deviation or error bars do not apply here. MM = multimodal, CH = cortical hubs, B = Blind, S = Sighted. **(C)** Graph based layouts of the three hierarchical networks in LB and CB subjects [size of nodes = degree of differential links (DDL)]. Due to normalization and scale proportions, both segments of panel **(C)** were scaled to aid visualization. (a) unimodal cortices; (b) multimodal integration cortices.

Of note, sensory-related masks obtained with functional connectivity based parcellations involve primary as well as closely interconnected unimodal association cortices. Therefore, it is frequently observed, for instance, that somatosensory and motor cortices or primary auditory and some perisylvian regions are indivisible with these techniques (Yeo et al., 2011). For simplicity, we refer to somatosensory and motor systems as somato-motor, and the wide auditory mask as auditory system. The multimodal and cortical hub masks were chosen from the characterization of the multimodal integration and cortical hub networks performed by Sepulcre et al. (2012). Note that multimodal integration regions, as exhibited in Figure 1A, include the dorsolateral prefrontal cortex (DLPFC; Brodmann area (BA) 46), frontal eye field (FEF; BA 8), ventral premotor/anterior insula (vPM/AI; BA 44, 45), Operculum Parietal (OP; BA 1, 2, 3, 43, 40), temporoparietal junction (TPJ; BA 39, 40), (SPC; BA 7), LOC (included in BA 19), and the supplementary motor area/dorsal anterior cingulate (SMA/dACC; BA 6 and 32 posterior aspect). These *a priori* parcellation schemes were selected to interrogate unimodal (e.g., visual, somato-motor, auditory), multimodal and cortical hub association networks. Using this approach, we calculated the total amount and spatial distribution of the DDL between unimodal and multimodal regions (Figure 1) and between and within multimodal and cortical hubs regions (Figure 2).

##### Local and Distant Hubs of Connectivity Changes

The FDR-corrected *t*-test matrices were also used to map local and distant functional connectivity changes in LB and CB subjects using a data-driven, voxel-level whole-brain method called *Local and Distant Connectivity Analysis* (Sepulcre et al., 2010). The physical segregation of local and distant functional connections was based on previous descriptions of the local and distant functional connectivity organization of the human brain (Sepulcre et al., 2010). Although the principles were similar, methodological differences arose due to tailored method steps for this study. For instance, in the original description of the method, the preferential brain locations of typical local and distant functional connectivity in individual subjects were computed and characterized, while in this analysis *t*-test matrices (not individual matrices) were used to describe the amount of local and distant functional connectivity changes occurring between groups. The *Local and Distant Connectivity Analysis* is formally described as follow:

$$DDL_{L}(i)=\sum_{j=1}^{n}d(i,j)\left[dist(i,j)<thr\right]$$

$$DDL_{D}(i)\;=\sum_{j=1}^{n}d(i,j)[dist(i,j)>thr]$$

where *DDL*_*L*_(*i*) represents the local connectivity change in the voxel *i* and *DDL*_*D*_(*i*) the distant connectivity. *n* represents the number of voxels and *d*(*i, j*) is equal to 1 if the blind subjects have higher number of significant links compared to controls, based on a *t*-test analysis with FDR correction and 0 otherwise. Finally *dist*(*i, j*) represents the distance between the voxel *i* and *i*. If this distance is lower than a specific threshold *thr* the connectivity is defined as local or distant if is higher.

The binarized FDR-corrected *t*-test matrices were used to count the number of differential links or DDL that a given voxel in the brain had to its immediate neighborhood (all DDL to surrounding voxels within 16 mm of distance; local-DDL maps) or outside that neighborhood (all DDL to voxels outside 16 mm of distance; distant-DDL maps). As previously reported, the optimal segregation of local and distant connections is achieved around 12 mm of neighborhood (Sepulcre et al., 2010). Given our voxel resolution, we selected a 16 mm segregation as the finest physical distance for our data. This process was done for every voxel included in the original mask of 5138 voxels. Therefore, for each voxel, the degree of enhanced and disrupted connectivity to its local/regional and distant/distributed level was obtained. The local-DDL and distant-DDL were studied in two statistical conditions: blind > sighted and sighted > blind. In summary, this study explicitly evaluated the functional connectivity changes or differential links that were taking place inside (local-DDL) and outside (distant-DDL) neighboring voxels at the group level (see a methodological diagram in Supplementary Figure S2).

#### Network and Cortical Visualization

Network visualization in Figure 2 was done using Pajek software (Nooy et al., 2004) and the Kamada and Kawai (Kamada and Kawai, 1989) energy layout of graphs. The Kamada and Kawai (1989) algorithm is a force layout method based on a network energy minimization procedure that takes into account the difference between geodesic and pair-wise shortest-path distances of nodes in the graph.

**Figure 3:**
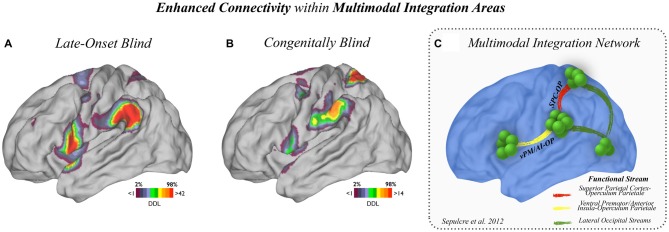
**Enhanced connectivity within multimodal regions. (A)** Enhanced connectivity within multimodal regions in LB and **(B)** CB subjects compared to MCs are displayed. LB subjects showed increased connectivity in the vPM/AI-OP stream, and CB subjects showed increased connectivity in the OP-SPC stream. **(C)** The main connectional axes involved in the multimodal integration network are displayed for reference.

**Figure 4:**
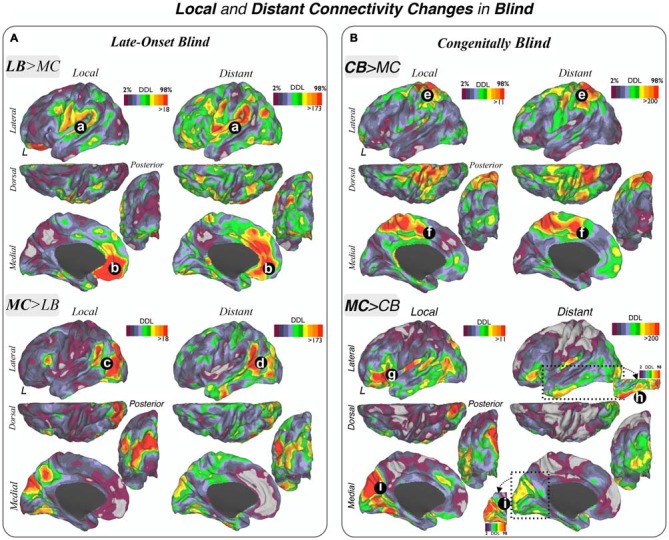
**Local and distant connectivity changes in blindness.** Increases and decreases in local and distant connectivity patterns in LB **(A)** and CB **(B)** subjects compared to their sighted MCs are displayed. **(A,B)**: (a) TPJ, OP/vPM; (b) medial prefrontal cortex; (c) MT+/lateral occipital areas; (d) temporal posterior/angular/parietal inferior cortex; (e) SPC; (f) medial SMA; (g) ventrolateral prefrontal cortex (VLPFC)/AI; (h) inferior temporal gyrus; (i) medial aspects of the primary visual areas V1, V2 and V3. LB = Late Onset Blind, CB = Congenitally Blind, MS = Matched Sighted controls.

Cortical data were visualized on the brain surface using the population-average, landmark- and surface-based (PALS) surface and plotted using CARET software (Van Essen, 2005). To aid visualization, all surface images are displayed using a normalized color scale (2–98% threshold of DDL). DDL values shown in the cortical maps reflect corrected and highly statistically significant connectivity changes. To aid interpretation of the cortical maps DDL values in cortical maps represent raw values of DDL (Figures 1, 3, 4) while bar graphs show normalized DDL, based on the size of the masks, in order to compare our interconnectivity findings in the same graph (Figures 1, 2). Note, results of the study are presented in the figures using only left hemisphere to avoid redundancy, but all study results including the right hemisphere are included as Supplementary Figure S4.

## Results

### Unimodal and Multimodal Interconnectivity in LB and CB Subjects Compared to their Matched Controls

The primary visual, auditory and somato-motor masks (displayed as different shades of red in Figure 1A) were used as *a priori* unimodal systems of interest to compare interconnectivity patterns between these unimodal cortices and multimodal integration areas (displayed in green), in LB, CB and MC subjects. As shown in the diagram of Figure 1A, there are three distinct theoretical outcomes: no change, increased or decreased functional interconnectivity in blind cohorts compared to their MCs.

As displayed in Figure 1B, we found that both enhanced and suppressed functional connections were present between modal and multimodal systems in LB and CB subjects. Blind subjects showed enhanced interconnectivity in the auditory and somato-motor areas to multimodal cortices compared to their MCs, along with less pronounced increased interconnectivity between visual and multimodal regions. In addition, both blind cohorts compared to their MCs exhibited a relative high number of suppressed links between visual regions and multimodal cortices, with less extensively reduced connectivity between somato-motor or auditory areas to multimodal regions. See Supplementary Figures S5 and S6 for a detailed account of increased and decreased cross-modal interconnectivity patterns across the unimodal cortices.

### Findings in Late-Onset Blind (LB)

#### Visual and Multimodal Integration Networks

Interconnectivity between unimodal visual areas and multimodal integration regions in LB subjects compared to MCs showed increased links in the TPJ and LOC, and decreased connectivity in the parieto-occipital junction, postcentral/supramarginal areas, and the most anterior aspects of the fusiform and lingual gyri (Figure 1C).

#### Auditory and Multimodal Integration Networks

Interconnectivity between auditory areas and multimodal integration regions in LB compared to MCs exhibited increased connectivity in the posterior region of the superior temporal gyrus/TPJ (Figure 1C). There were no comparative specific areas of reduced connectivity.

#### Somato-Motor and Multimodal Integration Networks

Interconnectivity between somato-motor areas and multimodal integration regions in LB compared to MCs demonstrated increased connectivity of the TPJ, an area of the motor cortex, and medial SMA (Figure 1C). There were no comparative specific areas of reduced connectivity.

### Findings in Congenitally Blind (CB)

#### Visual and Multimodal Integration Networks

Interconnectivity between visual areas and multimodal integration regions in CB compared to MC subjects showed areas of increased connectivity in the precuneus and the supramarginal cortex, along with decreased connectivity throughout visual cortex, particularly in lateral occipital regions (Figure 1D).

#### Auditory and Multimodal Integration Networks

Interconnectivity between auditory areas and multimodal regions in CB compared to MCs exhibited increased connectivity in the ventral areas of the somato-motor cortices and medial SMA, along with modest reduced connectivity in insular areas.

#### Somato-Motor and Multimodal Integration Network

Interconnectivity between somato-motor areas and multimodal integration regions in CB compared to MCs demonstrated increased connectivity in the SPC and medial SMA. There were no comparative specific areas of reduced connectivity.

#### Interconnectivity between Multimodal Integration Network and Cortical Hubs in Blind and Sighted Individuals

As showed in Figure 2A, sighted controls displayed a network layout in which the multimodal integration nodes serve as an interface between primary cortices and higher-order cortical hubs of the human brain. Following this observation, we extended our *Interconnector Analysis* method to study interconnectivity patterns and connectional relationships within and between multimodal integration and cortical hub nodes in blind individuals.

When comparing interactions across multimodal cortices and cortical hubs in LB and CB subjects compared to sighted controls, a high degree of connectivity was observed within multimodal regions (left-bottom column, Figure 2B) in comparison to other interconnectivity patterns such as multimodal regions to cortical hubs or cortical hub regions to one another. Consistent with this finding, multimodal integration regions in blind cohorts compared to MCs displayed only a small degree of suppressed links to one another, while interactions between cortical hubs and multimodal cortices or across cortical hub areas showed decreased interconnectivity (right-bottom column, Figure 2B). Figure 2C illustrates the node and network topological locations of enhanced connectivity in LB and CB networks.

### Increased Connectivity within Multimodal Integration Regions

In order to more precisely analyze the multimodal and association cortex connectivity changes described in Figures 1, 2, two complementary approaches were adopted. First, the areas within the multimodal integration network that displayed high amount of plastic degree of connectivity were identified. As shown in Figure 3, CB and LB subjects showed enhanced connections within multimodal regions compared to sighted controls; the LB group exhibited increased connectivity with the vPM/AI and OP (Figure 3A) while the CB cohort engaged OP and SPC regions (Figure 3B). The main connectional axes involved in the multimodal integration network are displayed for reference purposes in Figure 3 [based on Sepulcre et al. (2012)].

#### Local and Distant Connectivity Changes in Blind

Secondly, the DDL were investigated using a whole-brain data driven approach to characterize connectivity changes in LB and CB subjects that would not be detected by only investigating *a priori* unimodal, multimodal and cortical hubs systems (Figure 4). This DDL method delineated central brain areas with the highest number—or degree—of different functional connections between blind and sighted groups, and results were segregated into local and distant physical information in order to localize the main regional and distributed centers of connectivity changes.

The LB group compared to their MCs demonstrated a high degree of increased connectivity in multimodal integration regions including the TPJ, OP and vPM cortex in both local and distant maps (Figure 4A). The ventromedial prefrontal cortex (VMPFC) also showed increased local and distant connectivity in LB subjects, as did to a lesser extent other regions such as the intraparietal sulcus (IPS) and parieto-occipital junction. In addition, there was a high degree of reduced local and distant functional connections in the LB group compared to their MCs in lateral occipital regions (including the LO, MT+, V2, V3 and temporo-occipital junction (TOJ) areas). Interestingly, a portion of the TPJ posterior to areas displaying increased connectivity showed a high degree of reduced connectivity in the LB subjects. Moreover, when comparing the local and distant maps, we observed that the area posterior to the TPJ is predominantly reduced in distant connectivity while the reductions in lateral occipital area were predominantly local.

Using the same network analytical strategy, CB subjects compared to their MCs showed prominent enhanced local and distant connectivity in the SPC (Figure 4B). Additionally, the dorsal-medial cortical surface of the SMA and cingulate cortex exhibited increased connectivity. CB subjects exhibited diminished local connectivity in primary visual areas, particularly in V1, V2 and V3 (except for a focal portion of V1 posteriorly) and anterior and posterior insula. Decreased distant connectivity was also observed prominently in lateral, ventrolateral and ventromedial parts of the temporal and occipital lobes. The inferior frontal gyrus, pars orbitalis showed reduced local and distant connectivity in CB compared to their MCs.

## Discussion

We found that LB and CB subjects compared to MCs demonstrated enhanced functional connections that potentially underlie aspects of sensory adaptive phenomena after visual deprivation. Importantly, at the level of the multimodal integration network both blind cohorts exhibited increased interconnectivity across multimodal integration areas and between modal regions and multimodal integration cortices. Complementary data-driven analyses probing whole-brain local and distant functional connectivity patterns confirmed increased functional connectivity centered in multimodal integration cortices in blind subjects compared to sighted controls. These findings suggest that while functional alterations occurred in unimodal cortices including the LOC, extensive neuroplastic changes in blind individuals occurred at the level of multimodal and higher-order integrative processing regions.

The phenomenon of cross-modal neuroplasticity between unimodal cortices has been extensively demonstrated in sighted and blind subjects (Pascual-Leone et al., 2005). Visual deprivation results in cortical reorganization that appears to facilitate the processing of non-visual information and aids in overall perception, saliency and environmental characterization (Stein et al., 2001). It is also appreciated that cross-modal training can have a substantial impact on multisensory integration capabilities, suggesting a high degree of neuroplastic reorganization throughout the human life-span (Yu et al., 2010). For instance, the visual cortex is reversibly activated by tactile stimulation only 5 days after visual deprivation in sighted humans (Pascual-Leone and Hamilton, 2001), suggesting that competition between visual and non-visual afferents is normally present in the visual cortex. Such short-term effects are presumably not necessarily due to newly generated macroanatomical connections but rather reflect functional plasticity of existing connections, and have led to the formulation of theories like the *metamodal brain organization*, where brain regions function as operators of specific processes independent of the sensory input and they select out from the available sensory modalities the optimal one for a given operation (Pascual-Leone and Hamilton, 2001). So for example, in congenital, early and late acquired blind subjects, whole-brain imaging studies have consistently found functional adaptive changes centered in LOC areas, leading to the conceptualization of the LOC as a metamodal operator for shape (Amedi et al., 2007). In addition, activation of extrastriate areas has been demonstrated during non-visual tasks, such as during tactile spatial resolution (Van Boven et al., 2000), pitch processing (Gougoux et al., 2004), auditory spatial processing (Niemeyer and Starlinger, 1981; Lessard et al., 1998; Röder et al., 1999) and Braille reading (Sadato et al., 1996). Cortico-cortical pathways through which information from non-visual to visual areas is potentially transmitted (Mahon et al., 2009; Klinge et al., 2010), suggests that the cortical hierarchy in the occipital cortex may be reversed following visual deprivation (Büchel, 2003). While much emphasis has been given to elucidating cross-modal neuroplastic changes centered in the visual system, several important questions remained unsolved regarding cortical reorganization in blind subjects. A key issue of uncertainty pertained to the potential for broadly distributed network alterations occurring in the context of visual deprivation. It had been previously postulated that changes in brain activity seen in activation studies may be only delineating limited aspects of more extensive network reorganizations (Ricciardi et al., 2014). Consistent with such notions a recent study demonstrated the importance of association cortices facilitating multimodal integration at the level of the superior colliculus in an animal model (Yu et al., 2016).

The functional connectivity patterns that we found show complex and extensive interconnectivity changes in blind subjects as compared with sighted individuals. CB and LB subjects exhibited diminished local connectivity in primary and secondary visual areas, findings supported by previous works in functional connectivity analysis such as Qin and colleagues (Liu et al., 2007; Qin et al., 2015) as well as in structural analyses (Shimony et al., 2006; Leporé et al., 2010), identifying aspects of degenerative mechanisms following visual deprivation. Moreover, we found robust increases in interconnectivity between multimodal integration cortices and auditory and somato-motor systems (and to a lesser degree with non-primary visual cortices) in both LB and CB subjects. In this sense, the finding of enhanced interconnectivity of somato-motor and auditory systems to multimodal integration areas, along with increased interconnectivity across multimodal integration areas, may constitute a neural substrate of heightened auditory and somatosensory abilities in blind subjects (Voss et al., 2010).

The most extensive cortical network changes in our blind cohorts as compared to sighted controls occurred in high-order integration zones, such as multimodal integration areas in the TPJ, supramarginal gyrus (SMG) and superior parietal lobe. As previously postulated, the strength of multisensory integration may be based on independent and temporally aligned signals derived from primary stimuli converging onto multisensory neurons (Wallace et al., 2004). Our results support that view and show that adaptive neuroplastic changes occurring after a sensory deprivation are particularly impacting areas of multisensory convergence rather than regions of predominantly unimodal processing. Furthermore, cross-modal phenomenon may be made possible, in part, through common relay stations facilitating the union between the sensory modalities (rather than only through cortical-cortical connections across unimodal cortices). It has been previously described that visual information runs along functional streams in the human brain (Goodale and Milner, 1992; Van Essen et al., 1992; Rizzolatti and Matelli, 2003). The primary visual cortex connects with extrastriate (LOC), MT+, SPL and OP regions, and subsequently converges onto core regions of the multimodal network, particularly the OP-vPM/AI connectivity axes (Figure 3; Sepulcre et al., 2012). Our interconnectivity analysis of the multimodal integration system showed that CB subjects had prominent increased functional connections in the SPC and OP regions, while LB exhibited increased functional connections with the OP-TPJ and vPM/AI regions. These findings support the interpretation that early-onset blindness may lead to functional neuroplastic alterations in earlier connectional axes of the visual and multimodal integration streams, while LB may impact primarily upstream multimodal processing areas, such as the ventral system of OP-vPM/AI connections.

In addition to increased connectivity within the TPJ and adjacent regions in LB subjects and within the SPC in CB subjects compared to controls, the dACC/middle cingulate cortex is another multimodal integration region that showed enhanced functional connectivity in blind subjects (particularly CB) compared to controls. Meta-analyses in sighted subjects have identified the anterior middle cingulate cortex as an integrative center for negative emotion, visceral-somatic nociceptive processing and cognitive control (Shackman et al., 2011). Apart from the cingulate gyrus, the VMPFC, a region implicated in the modulation of emotion, visceral-somatic processing and self-awareness (Ongür and Price, 2000), displayed significantly increased regional (local) degree of connectivity in LB subjects. The VMPFC is also known to be interconnected to the hypothalamus and brainstem as part of the interoceptive system mediating bodily reactions and internal mood states (Ongür and Price, 2000). The findings of enhanced connectivity within the cingulate gyrus and VMPFC perhaps reflect the potential for improved processing of self-related internal (emotional and bodily) states in blind subjects.

There are a few limitations to this study. First, this fcMRI study only utilized BOLD signal as the measure of brain connectivity and therefore should be complemented by future whole-brain structural analyses to fully capture the richness of brain and synaptic rewiring. BOLD-based fcMRI measures intrinsic activity correlations between brain regions that seem to be sufficiently constrained by anatomy to reveal informative estimates of connectivity properties (Biswal et al., 1995; Van Dijk et al., 2010). However, it is important to emphasize that fcMRI can reflect mono- and polysynaptic connectivity, thus, it should not be considered a direct measure of monosynaptic or tract anatomical connectivity. Secondly, this study did not allow for direct comparison of LB and CB subjects due to differences in the MRI protocol acquisition. In addition, this study aimed to investigate neuroplastic re-organization in multimodal integration regions, but did not seek to detect potential lateralized network effects nor specific associations between the degree of neuroplastic alterations in multimodal integration regions and auditory/somatomotor abilities which can be further investigated in larger sample sized neuroimaging studies. While more studies are needed to elucidate detailed network differences between blind cohort subtypes (including the future use of other complementary graph theory based techniques such as a weighted-degree analysis), our findings offer insights into the central role of shifts in connectivity with multimodal integration cortices in the large-scale neural reorganization in blind subjects.

## Conclusion

In conclusion, we used two complementary graph theory based network approaches to elucidate whole-brain functional connectivity reorganizational changes in LB and CB cohorts each compared to MC subjects. While functional network alterations occurred in sensory cortices, neuroplastic changes were most prominent and centralized within multimodal and association cortices. LB subjects compared to sighted controls showed enhanced multimodal integration connections as measured by DDL values in the parieto-opercular, TPJ and vPM regions, while CB individuals compared to sighted controls exhibited increased SPC connections. This study reveals the critical role of recipient multi-sensory integration areas in network reorganization and cross-modal plasticity in blind individuals.

## Author Contributions

LO-T: designed experiments, analyzed data, interpreted findings and drafted the manuscript. TO: designed experiments, interpreted the findings and drafted the manuscript. DLP: interpreted the findings and drafted the manuscript. JIA: designed experiments, collected data and critically reviewed the manuscript. ID: analyzed data and drafted the manuscript. AP-L: designed experiments, interpreted results and critically reviewed the manuscript. JS: designed experiments, developed analytical tools, analyzed data, interpreted the findings and drafted the manuscript. All authors discussed the results, reviewed and edited the manuscript and approved the submitted version.

## Funding

This work was supported by the Fundación Mutua Madrileña (4131220 to LO-T and TO) The National Institute of Biomedical Imaging and Bioengineering (1K23EB019023-01RM to JS), the National Institutes of Health (R01HD069776, R01NS073601, R21 MH099196, R21 NS082870, R21 NS085491, R21 HD07616 to AP-L), the Berenson-Allen Foundation (to AP-L), the Sidney R. Baer Jr. Foundation (to AP-L), and the Harvard Catalyst | The Harvard Clinical and Translational Science Center (NCRR and the NCATS NIH, UL1 RR025758 to AP-L).

## Conflict of Interest Statement

AP-L serves on the scientific advisory boards for Nexstim, Neuronix, Starlab Neuroscience, Allied Mind, Neosync, Magstim, Axilum Robotics, and Novavision and is listed as an inventor on several issued and pending patents on the real-time integration of transcranial magnetic stimulation (TMS) with electroencephalography (EEG) and magnetic resonance imaging (MRI). All other authors declare no potential conflicts of interest with respect to the research, authorship and publication of this article.
