# Supplementary material for: Brain Plasticity in Blind Subjects Centralizes Beyond the Modal Cortices
Source: Front Syst Neurosci. 2016 Jul 8;10:61. doi: 10.3389/fnsys.2016.00061 (PMC4937754; doi:10.3389/fnsys.2016.00061)
Supplement: Supplementary file 1 [file DataSheet_1.doc]

**Supporting Information Figure Legends**

**Fig. S1. *Cross-modal activation foci in whole-brain analytical studies.***  Display of activation foci from functional imaging studies performed in late-onset blind (LB) and congenitally blind (CB) subjects in whole brain analyses without *a priori* regions of interest(Amedi et al., 2003; Arnaud et al., 2013; Bedny et al., 2011, 2012; Büchel et al., 1998; Burton et al., 2002, 2004; Collignon et al., 2011; Gizewski et al., 2003; Gougoux et al., 2009; Ma and Han, 2011; Matteau et al., 2010; Ptito et al., 2005; Ricciardi et al., 2007, 2009; Sadato et al., 2002; Striem-Amit et al., 2012; Voss et al., 2008, 2011; Watkins et al., 2012). L indicates left; R, right.

**Fig. S2. *Graphic depiction of statistical analysis preprocessing steps.*** Graphic depiction of statistical analysis preprocessing steps. **(I)** A voxel by time association matrix was computed for each individual. **(II)** A voxel by voxel association matrix was determined for each group. **(III)** Statistical parametrical analysis (FDR-corrected T-test) between blind groups and their matched controls were performed. **(IV)** Local degree of differential links and **(V)** distant degree of differential links were calculated.

**Fig. S3. *Seed based analysis.***   Seed based analysis located in the inferior-lateral occipital cortex was performed to confirm that our pipeline approach was equivalent to prior investigations with congenitally blind subjects as reported in Bedny, 2011.

**Fig. S4. *Local and distant functional connectivity changes.***  Local and distant functional connectivity changes in both hemispheres of the late-onset blind (LB) **(a)** and congenitally blind (CB) **(b)** subjects compared to their matched sighted controls (MC). L indicates left; R, right.

**Fig. S5.** ***Increased Cross-modal Interconnectivity in Blindness***. (**A**) Unimodal cortices (visual, auditory and somato-motor), represented as different shades of red, were used as *a priori* regions of interest, in both late onset (LB) and congenitally blind (CB) subjects compared to their matched sighted controls (MC), to characterize enhanced interconnectivity between them. (**B**) Bar graphs show statistically significant increased interconnectivity between unimodal cortices in blind subjects compared to matched controls. These results were computed using the DDL comparison analysis at the group level, and thus the variance of the data at the individual subject level is not captured. V=visual, A=auditory, SM=somato-motor. Panels **C** and **D** present interconnectivity patterns in LB and CB subjects compared to their MCs respectively. **(a)** lateral occipital cortex (LOC); **(b)** posterior aspect of the superior temporal gyrus; **(c)** precuneus; **(d)** superior parietal cortex; **(e)** ventral premotor cortex/motor cortex**.**

**Fig. S6. *Decreased Cross-modal Interconnectivity in Blindness.*** Unimodal cortices were used as *a priori* regions of interest, in both late onset (LB) and congenitally blind (CB) subjects compared to theirs matched sighted controls (MC), to characterize decreased interconnectivity between them. (**A**) Bar graphs show statistically significant decreased interconnectivity between unimodal cortices in blind subjects compared to matched controls. These results were computed using the DDL comparison analysis at the group level, and thus the variance of the data at the individual subject level is not captured. V=visual, A=auditory, SM=somato-motor. Panels **B** and **C** present interconnectivity patterns in LB and CB subjects compared to their MCs respectively. **(a)** lateral occipital cortex (LOC); **(b)** ventral premotor cortex; **(c)** primary visual cortex; **(d)** superior parietal cortex; **(e)** ventral premotor cortex/motor cortex; **(f)** motor cortex; **(g)** insula.

**Supplementary References**

Amedi, A., Raz, N., Pianka, P., Malach, R., and Zohary, E. (2003). Early “ visual ” cortex activation correlates with superior verbal memory performance in the blind. 6, 758–766.

Arnaud, L., Sato, M., Ménard, L., and Gracco, V. L. (2013). Repetition Suppression for Speech Processing in the Associative Occipital and Parietal Cortex of Congenitally Blind Adults. *PLoS One* 8, e64553. doi:10.1371/journal.pone.0064553.

Bedny, M., Caramazza, a, Pascual-Leone, a, and Saxe, R. (2012). Typical neural representations of action verbs develop without vision. *Cereb. Cortex* 22, 286–93. doi:10.1093/cercor/bhr081.

Bedny, M., Pascual-Leone, A., Dodell-Feder, D., Fedorenko, E., and Saxe, R. (2011). Language processing in the occipital cortex of congenitally blind adults. *Proc. Natl. Acad. Sci. U. S. A.* 108, 4429–4434. doi:10.1073/pnas.1014818108.

Büchel, C., Price, C., Frackowiak, R. S., and Friston, K. (1998). Different activation patterns in the visual cortex of late and congenitally blind subjects. *Brain* 121 ( Pt 3, 409–419.

Burton, H., Sinclair, R. J., and McLaren, D. G. (2004). Cortical activity to vibrotactile stimulation: an fMRI study in blind and sighted individuals. *Hum. Brain Mapp.* 23, 210–228. doi:10.1002/hbm.20064.

Burton, H., Snyder, a Z., Diamond, J. B., and Raichle, M. E. (2002). Adaptive changes in early and late blind: a FMRI study of verb generation to heard nouns. *J. Neurophysiol.* 88, 3359–3371. doi:10.1152/jn.00129.2002.

Collignon, O., Vandewalle, G., Voss, P., Albouy, G., Charbonneau, G., Lassonde, M., et al. (2011). Functional specialization for auditory-spatial processing in the occipital cortex of congenitally blind humans. *Proc. Natl. Acad. Sci. U. S. A.* 108, 4435–4440. doi:10.1073/pnas.1013928108.

Gizewski, E. R., Gasser, T., De Greiff, a., Boehm, a., and Forsting, M. (2003). Cross-modal plasticity for sensory and motor activation patterns in blind subjects. *Neuroimage* 19, 968–975. doi:10.1016/S1053-8119(03)00114-9.

Gougoux, F., Belin, P., Voss, P., Lepore, F., Lassonde, M., and Zatorre, R. J. (2009). Voice perception in blind persons: A functional magnetic resonance imaging study. *Neuropsychologia* 47, 2967–2974. doi:10.1016/j.neuropsychologia.2009.06.027.

Ma, Y., and Han, S. (2011). Neural representation of self-concept in sighted and congenitally blind adults. *Brain* 134, 235–246. doi:10.1093/brain/awq299.

Matteau, I., Kupers, R., Ricciardi, E., Pietrini, P., and Ptito, M. (2010). Beyond visual, aural and haptic movement perception: hMT+ is activated by electrotactile motion stimulation of the tongue in sighted and in congenitally blind individuals. *Brain Res. Bull.* 82, 264–270. doi:10.1016/j.brainresbull.2010.05.001.

Ptito, M., Moesgaard, S. M., Gjedde, A., and Kupers, R. (2005). Cross-modal plasticity revealed by electrotactile stimulation of the tongue in the congenitally blind. *Brain* 128, 606–614. doi:10.1093/brain/awh380.

Ricciardi, E., Bonino, D., Sani, L., Vecchi, T., Guazzelli, M., Haxby, J. V, et al. (2009). Do we really need vision? How blind people “see” the actions of others. *J. Neurosci.* 29, 9719–9724. doi:10.1523/JNEUROSCI.0274-09.2009.

Ricciardi, E., Vanello, N., Sani, L., Gentili, C., Scilingo, E. P., Landini, L., et al. (2007). The effect of visual experience on the development of functional architecture in hMT+. *Cereb. Cortex* 17, 2933–9. doi:10.1093/cercor/bhm018.

Sadato, N., Okada, T., Honda, M., and Yonekura, Y. (2002). Critical period for cross-modal plasticity in blind humans: a functional MRI study. *Neuroimage* 16, 389–400. doi:10.1006/nimg.2002.1111.

Striem-Amit, E., Cohen, L., Dehaene, S., and Amedi, A. (2012). Reading with Sounds: Sensory Substitution Selectively Activates the Visual Word Form Area in the Blind. *Neuron* 76, 640–652. doi:10.1016/j.neuron.2012.08.026.

Voss, P., Gougoux, F., Zatorre, R. J., Lassonde, M., and Lepore, F. (2008). Differential occipital responses in early- and late-blind individuals during a sound-source discrimination task. *Neuroimage* 40, 746–758. doi:10.1016/j.neuroimage.2007.12.020.

Voss, P., Lepore, F., Gougoux, F., and Zatorre, R. J. (2011). Relevance of spectral cues for auditory spatial processing in the occipital cortex of the blind. *Front. Psychol.* 2, 48. doi:10.3389/fpsyg.2011.00048.

Watkins, K. E., Cowey, A., Alexander, I., Filippini, N., Kennedy, J. M., Smith, S. M., et al. (2012). Language networks in anophthalmia: maintained hierarchy of processing in “visual” cortex. *Brain* 135, 1566–77. doi:10.1093/brain/aws067.
