# Supplementary figures and images for: Brain Plasticity in Blind Subjects Centralizes Beyond the Modal Cortices
Source: Front Syst Neurosci. 2016 Jul 8;10:61. doi: 10.3389/fnsys.2016.00061 (PMC4937754; doi:10.3389/fnsys.2016.00061)

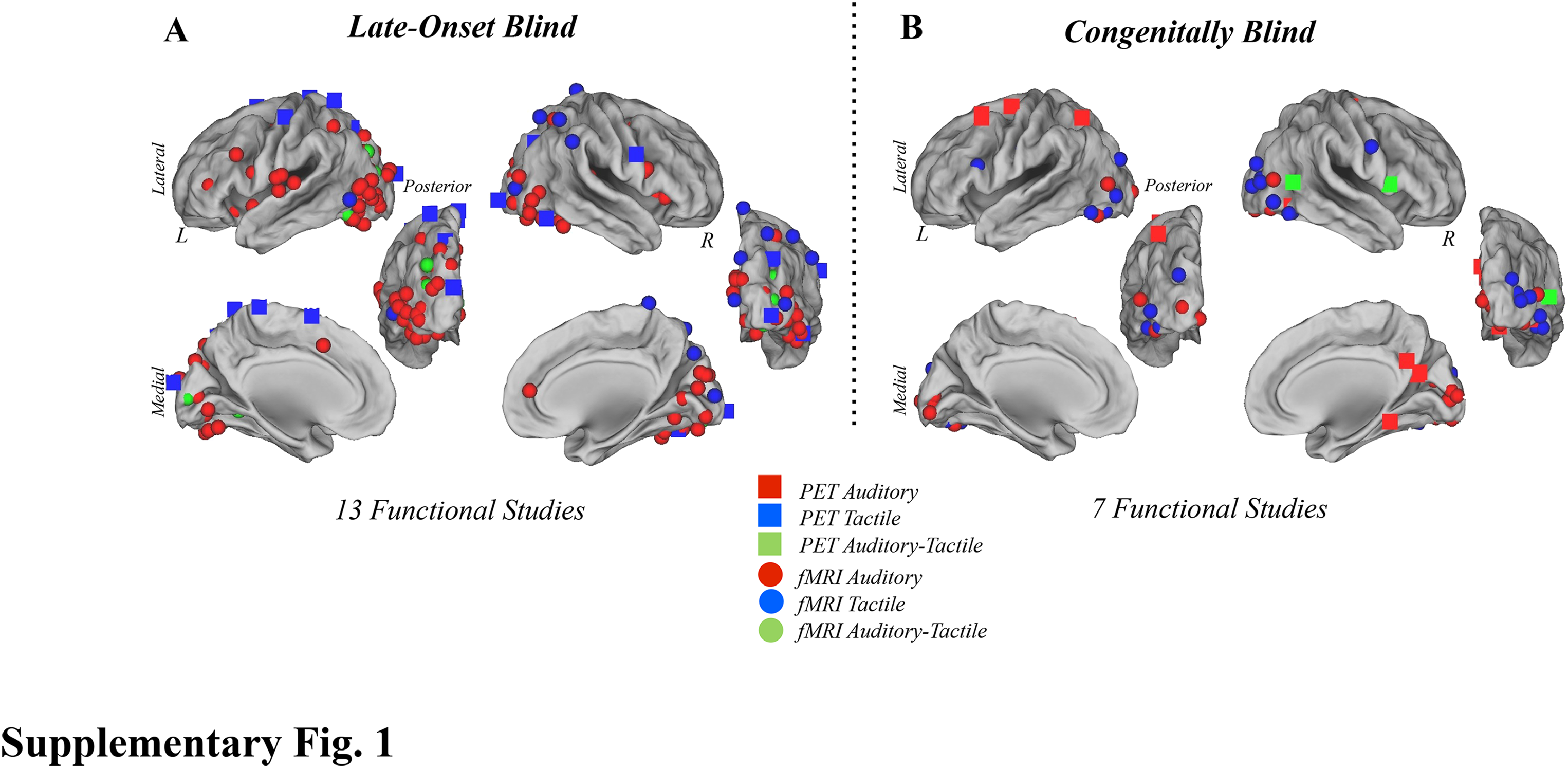

Supplement: Supplementary file 2 [file Image_1.tif]

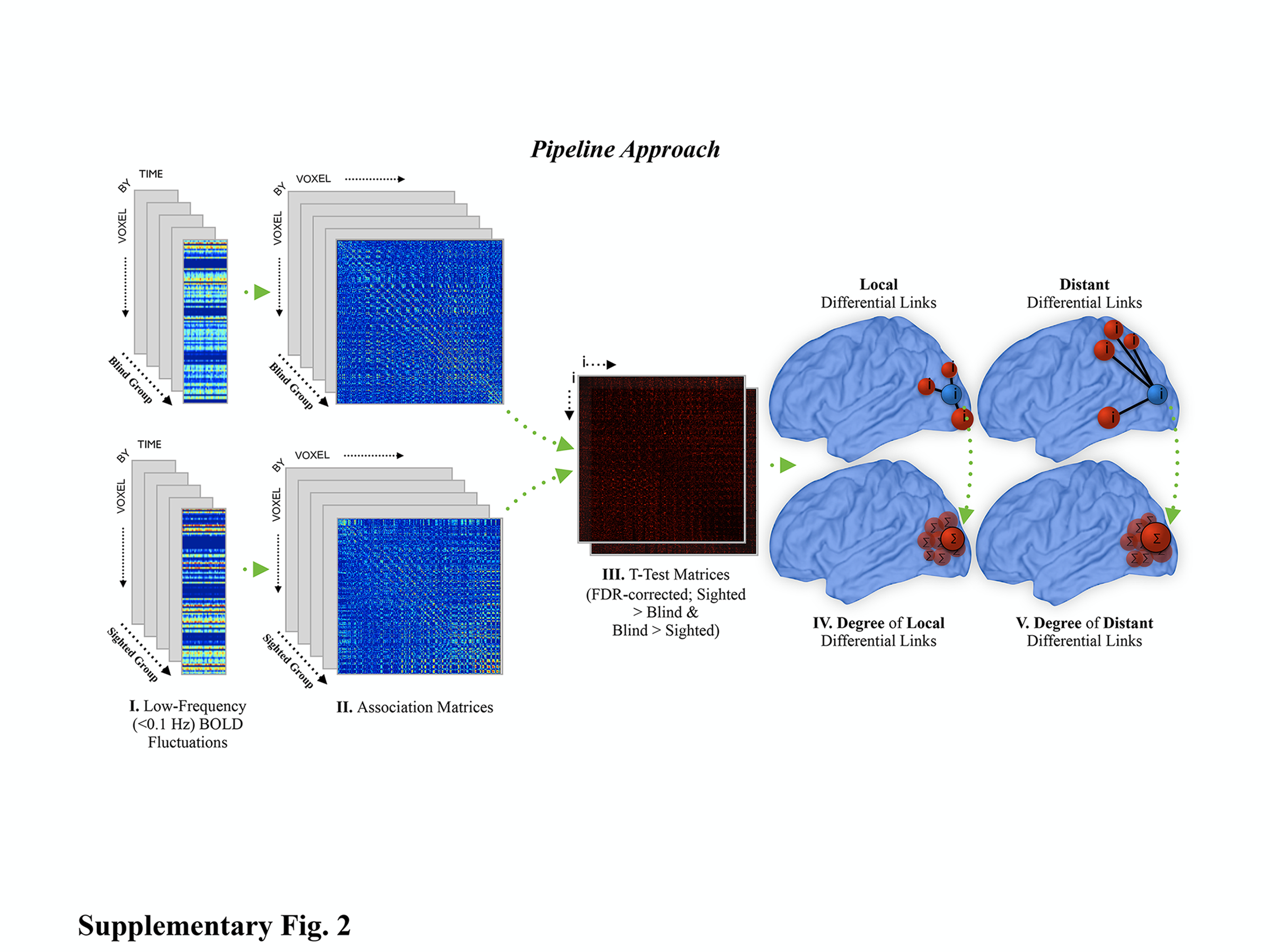

Supplement: Supplementary file 3 [file Image_2.tif]

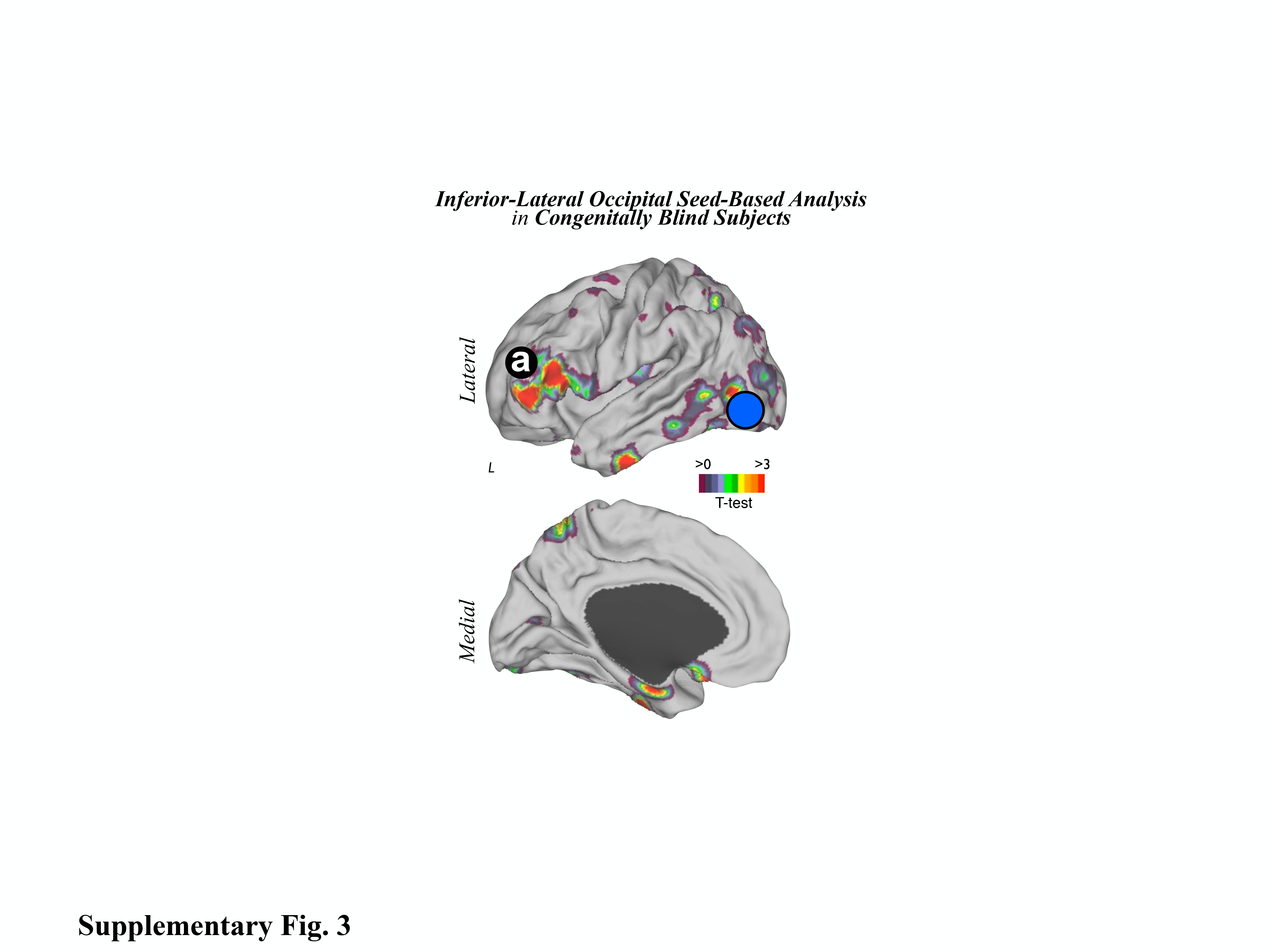

Supplement: Supplementary file 4 [file Image_3.tif]

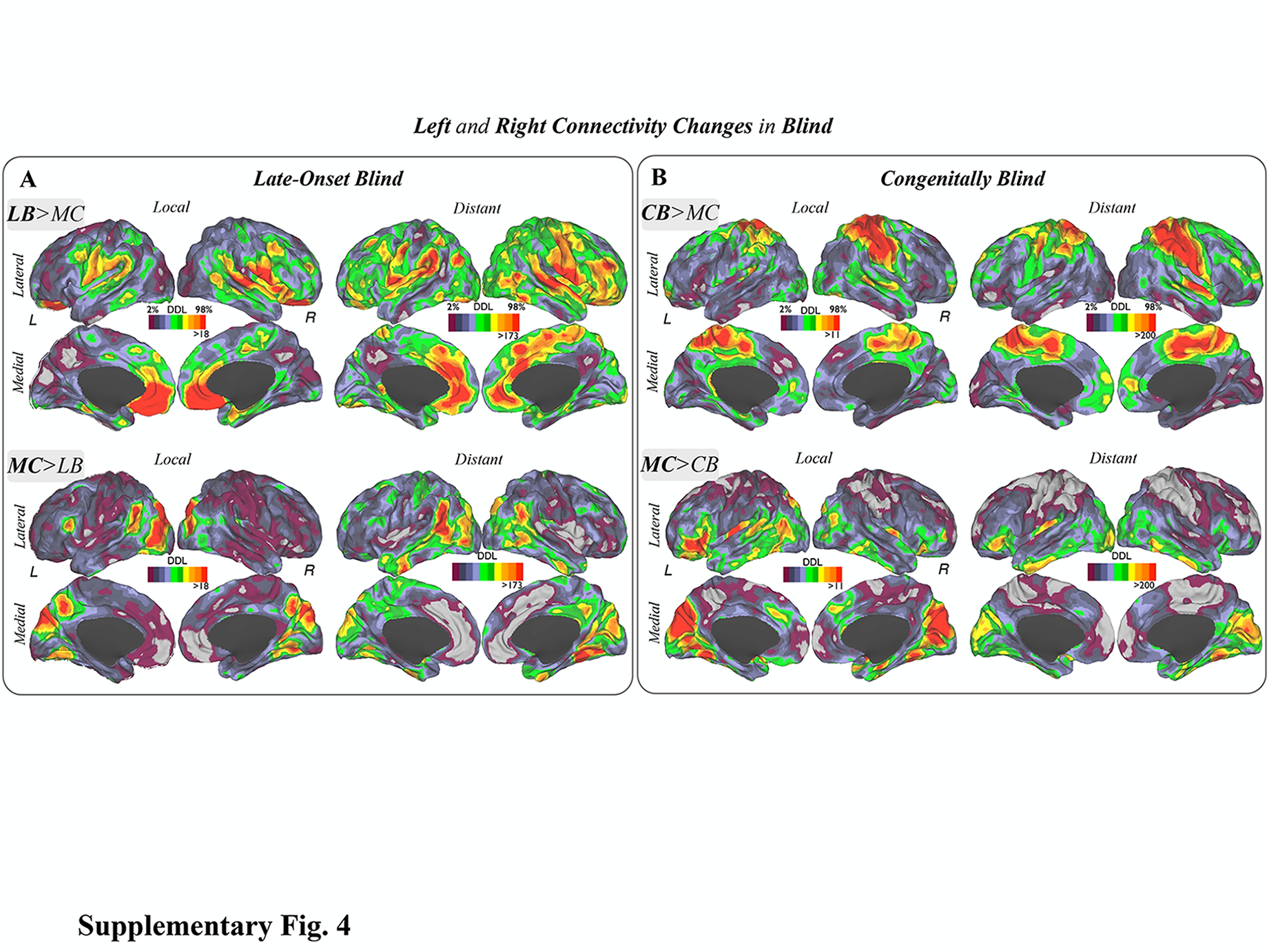

Supplement: Supplementary file 5 [file Image_4.tif]

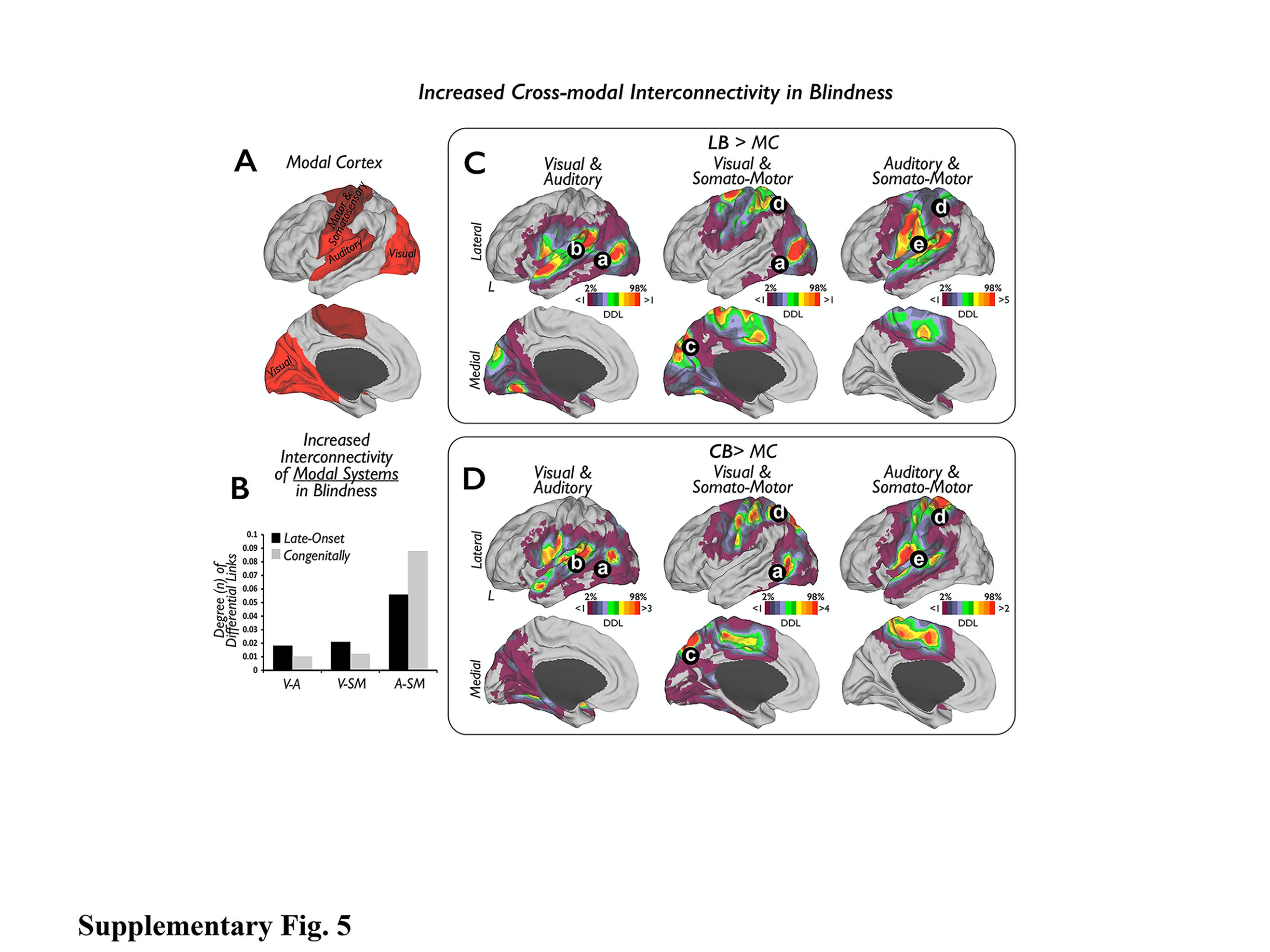

Supplement: Supplementary file 6 [file Image_5.tif]

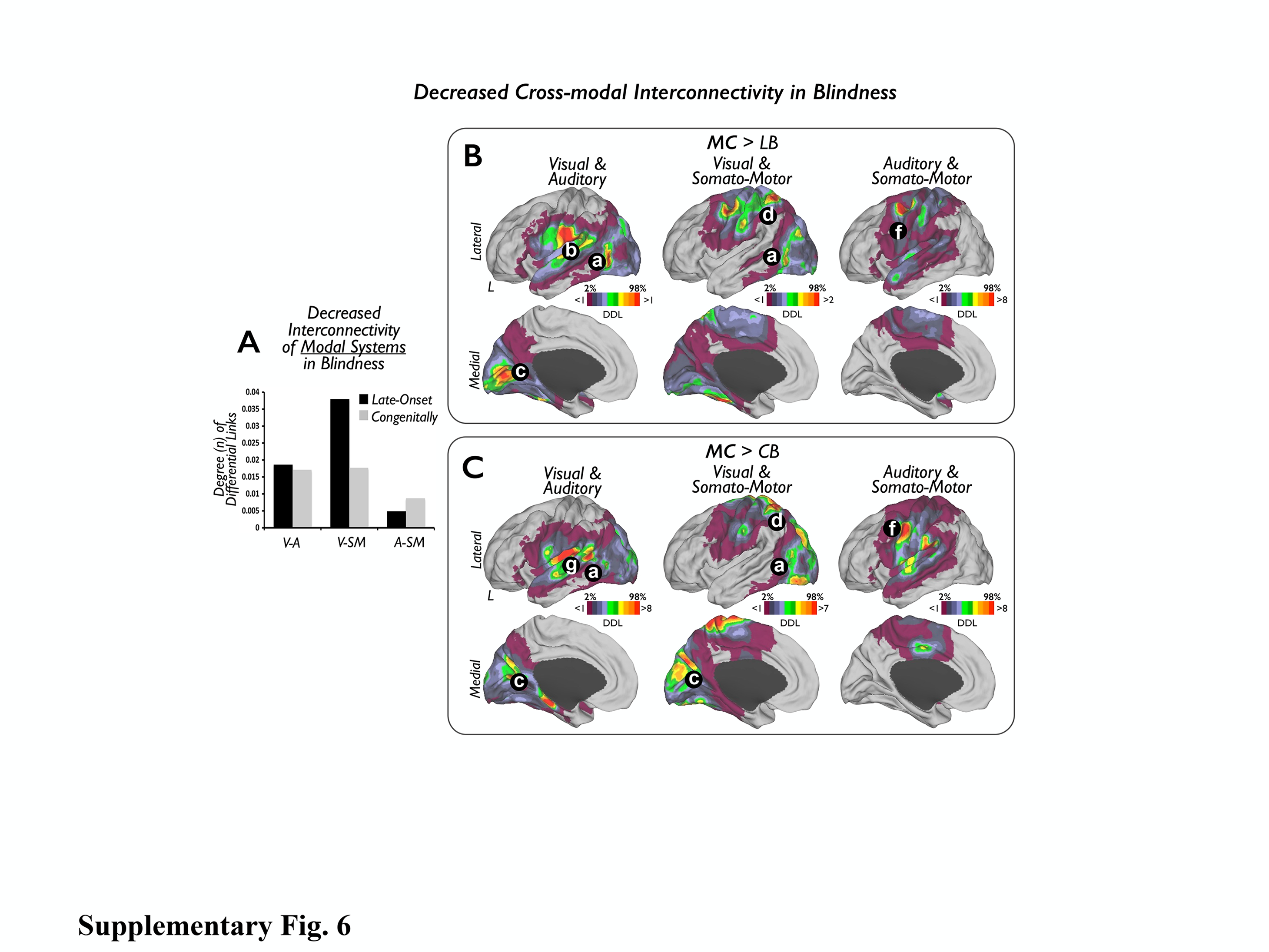

Supplement: Supplementary file 7 [file Image_6.tif]
